# Isolated Renal Artery Dissection: A Systematic Review of Case Reports

**DOI:** 10.7759/cureus.6960

**Published:** 2020-02-11

**Authors:** Anil Jha, Maxwell Afari, Ioannis Koulouridis, Tariq Bhat, Lawrence Garcia

**Affiliations:** 1 Internal Medicine, Lawrence General Hospital, Lawrence, USA; 2 Cardiology, Maine Medical Center, Boston, USA; 3 Cardiology, St. Elizabeth Medical Center, Brighton, USA; 4 Cardiology, St. Elizabeth's Medical Center / Tufts University School of Medicine, Brighton, USA

**Keywords:** isolated renal artery dissection, systematic review, renovascular hypertension, fibromuscular dysplasia, abdominal pain

## Abstract

Isolated renal artery dissection (IRAD) is a rare and often unrecognized clinical entity, with a paucity of data on its epidemiology and management. We extracted 129 cases of IRAD from the medical literature between 1972 and 2016. IRAD as a result of an extended dissection from the aorta and splanchnic or mesenteric arteries was excluded.

The mean age of presentation was 42.7±12.9 years, with a male predominance (79%). Abdominal pain (75.9%) was the most common presenting symptom. Etiology was more likely to be spontaneous (76%) than traumatic (12%), iatrogenic (9%), or drug-induced (1.5%). The most common risk factors were hypertension (28.7%), fibromuscular dysplasia (8.5%), and Ehlers-Danlos syndrome (5.4%). Unilateral renal artery dissection (right 45.5%, left 40.5%) was more frequent than bilateral (14%). More than half (56.6%) of the cohort were managed medically (blood pressure control and /or anticoagulation). Of those who underwent intervention, endovascular stenting or embolization (35%) was utilized more frequently than nephrectomy or bypass (21%). Computed tomography (CT) and magnetic resonance angiography (MRA) have the highest diagnostic sensitivity (91% and 93%, respectively) as compared to ultrasonography (27%).

A high degree of clinical suspicion is required to diagnose IRAD. CT and MRI have a higher diagnostic sensitivity. As compared to invasive management, conservative management has comparable outcomes.

## Introduction and background

Isolated renal artery dissection (IRAD) was first reported in 1944 [1]. This condition is rare and only accounts for 1%-2% of all arterial dissections. In the majority of cases of IRAD, the etiology is unknown and, hence, most of them are classified as spontaneous. Iatrogenic injury, fibromuscular dysplasia, severe atherosclerosis, malignant hypertension, and connective tissue disorders have been associated with IRAD [2-4].

The clinical presentation of IRAD can be easily confused with other gastrointestinal (GI) and genitourinary (GU) conditions. It is possible that the advent of advanced imaging and diagnostic techniques could have made the diagnosis of IRAD timelier; however, this has not been investigated. There is a lack of robust clinical data on the characteristics of patients with IRAD and, currently, there are no clinical guidelines on the management of IRAD. This review sought to retrospectively evaluate all the published cases of IRAD so that its clinical characteristics, presentations, and treatment modalities could be further defined.

## Review

Method

An English-language systematic review of the literature of cases and case series of patients with IRAD was done. PUBMED, Google Scholar, Scopus, Web of Science, and EMBASE were searched using the terms renal artery, dissection, abdominal pain, and renovascular hypertension. Cases of IRAD due to an extended dissection from the aorta and splanchnic or mesenteric arteries were excluded. One author (MEA) screened titles and abstracts of database records and retrieved full texts for an eligibility assessment. Two authors (AKJ and MEA) independently extracted data from these cases. Discrepancies were resolved by consensus between both reviewers. The following data were extracted: demographics, year of publication, risk factors (renal artery aneurysm or stenosis, hypertension, fibromuscular dysplasia, Ehlers-Danlos syndrome, and polyarteritis nodosa), diagnostic tools, management strategy, and outcomes.

Statistical analyses were performed using the R statistical software package, version 2.15.2 (2012) (R Foundation for Statistical Computing, Vienna, Austria) along with the “caret” package. Binary variables are presented as percentages; Gaussian continuous variables are presented as mean ± standard deviation (SD) while non-Gaussian continuous variables are presented as median with interquartile range (IQR). Logistic regression was used to measure the association between the different imaging modalities and renal angiogram. Spearman correlation was used to investigate an association between year of publication and the age of IRAD presentation. The Welch two-sample t-test was used to investigate an association between gender and age upon presentation with IRAD. All analyses were two-sided using a p-value of ≤ 0.05 as the cutoff for statistical significance.

Results

**Figure 1:**
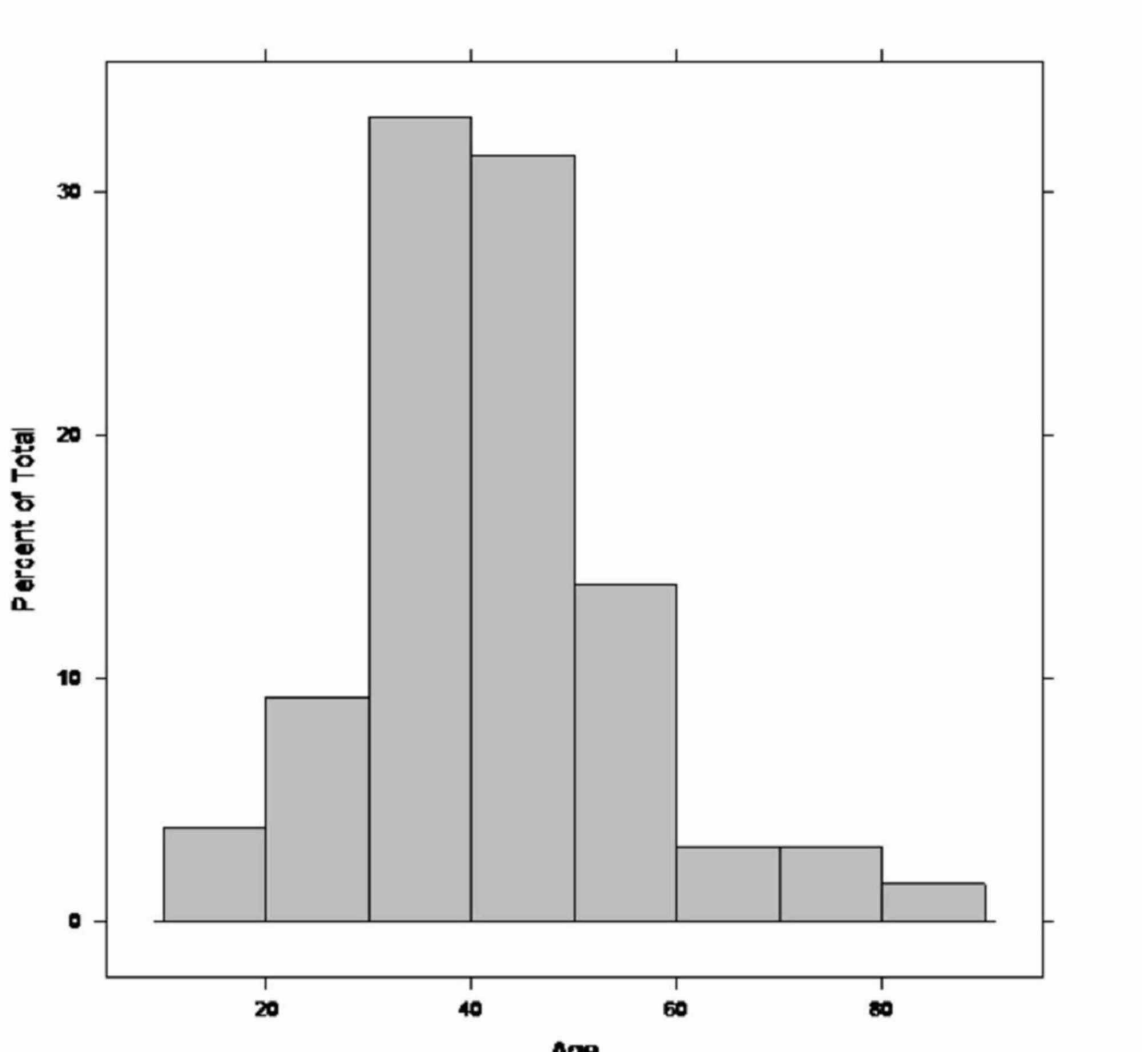
Histogram showing the distribution of age upon presentation with renal artery dissection The mean age is 43±13 years.

A total of 129 cases of renal artery dissection published between 1972 and 2016 were reviewed. The baseline characteristics of the included cohort are summarized in Table 1. The mean age of presentation was 42.7±12.9 years (Figure 1) with a predominance of the male gender (79%). Half of the cohort had a history of hypertension (54%) while fibromuscular dysplasia (8.5%) and Ehlers-Danlos syndrome (5.4%) were the other most commonly identified risk factors.

**Table 1:** Baseline characteristics of the included cohort

Year of publication, median (interquartile range)	2007 (2000, 2012)
Age, mean (SD)	43 (13)
Etiology of renal artery dissection, %	
Cocaine-induced	1.5
Iatrogenic	9.0
Spontaneous	76
Traumatic	12
Physical exertion	1.5
Sudden pain on presentation, %	88
Abdominal pain on presentation, %	76
Hematuria on presentation, %	15.4
Laterality of renal artery dissection, %	
Left	40.5
Right	45.5
Bilateral	14
Hypertension, %	54
Costovertebral angle tenderness on examination, %	28
Abdominal tenderness on examination, %	35
Presence of blood in the urinalysis, %	28
Treatment with anticoagulation, %	45
Treatment with antihypertensives, %	69
Surgical treatment, %	21
Endovascular stenting, %	35
Outcome, %	
Bad/poor prognosis (renal function didn’t improve)	8
Death	1
Good outcome (renal function improved)	91

The etiology of IRAD was more commonly spontaneous (76%) than traumatic (12%), iatrogenic (9%), due to inhaled cocaine use (1.5%), or due to extreme physical exertion (1.5%). Abdominal pain was the most common complaint (75.9%) while part of the cohort presented with hematuria (15.4%). Unilateral IRAD (right 45.5%, left 40.5%) was more commonly seen than bilateral (14%). Abdominal or kidney, ureter, and bladder (KUB) X-rays were used in only two patients in our review, with no abnormality found. The sensitivity of computerized tomography angiography (CTA) and magnetic resonance angiography (MRA) were better (91% and 93%), respectively, as compared to Doppler ultrasound (27%).

**Figure 2:**
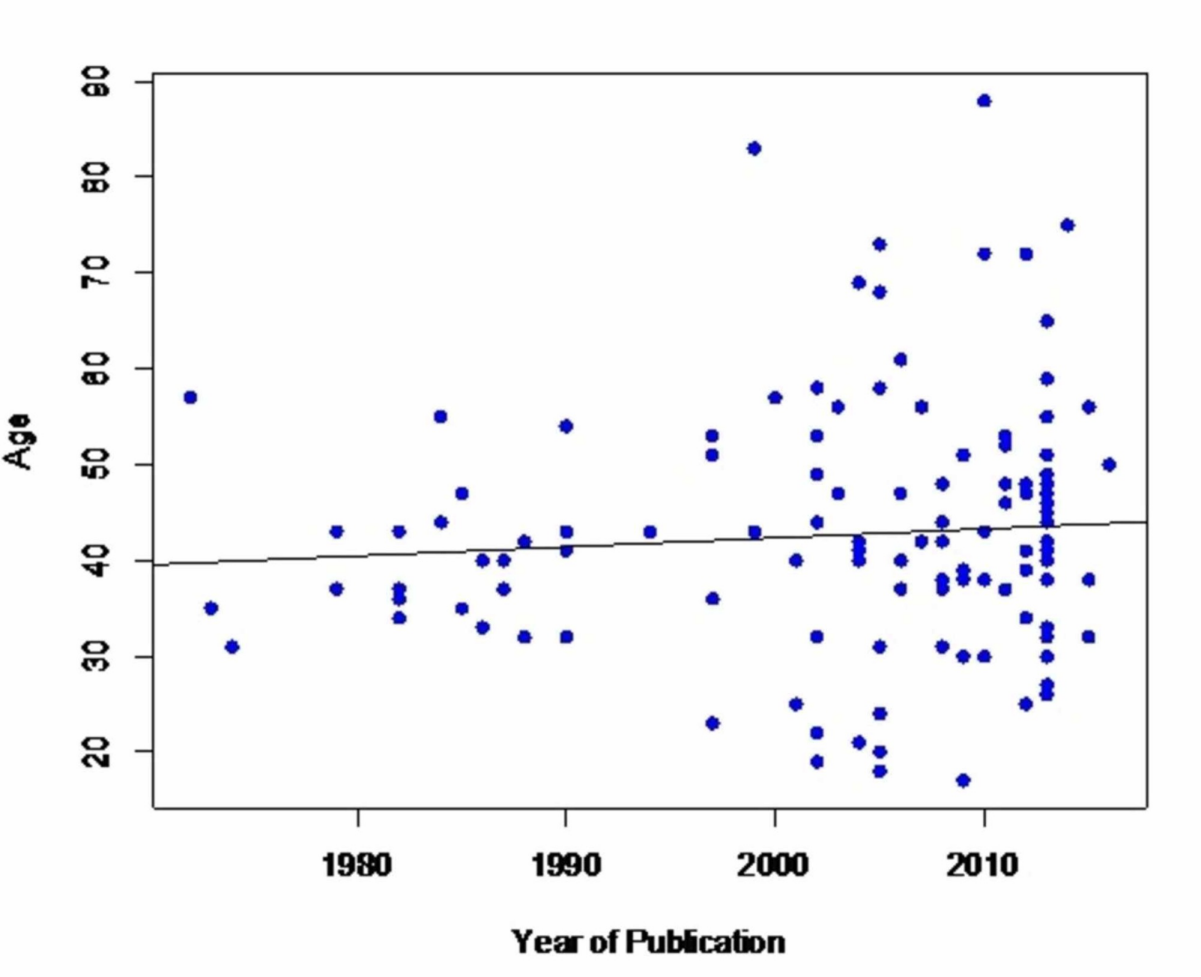
Scatterplot showing the relationship between the age of presentation with renal artery dissection and year of publication The average year of presentation did not change significantly over the years. Spearman’s rank correlation p-value is 0.419 and Rho (which is a measure of the quantification of the association) is 0.07, which is considered low. The black line represents an unadjusted linear regression; the slightly positive slope is non-significant statistically.

**Figure 3:**
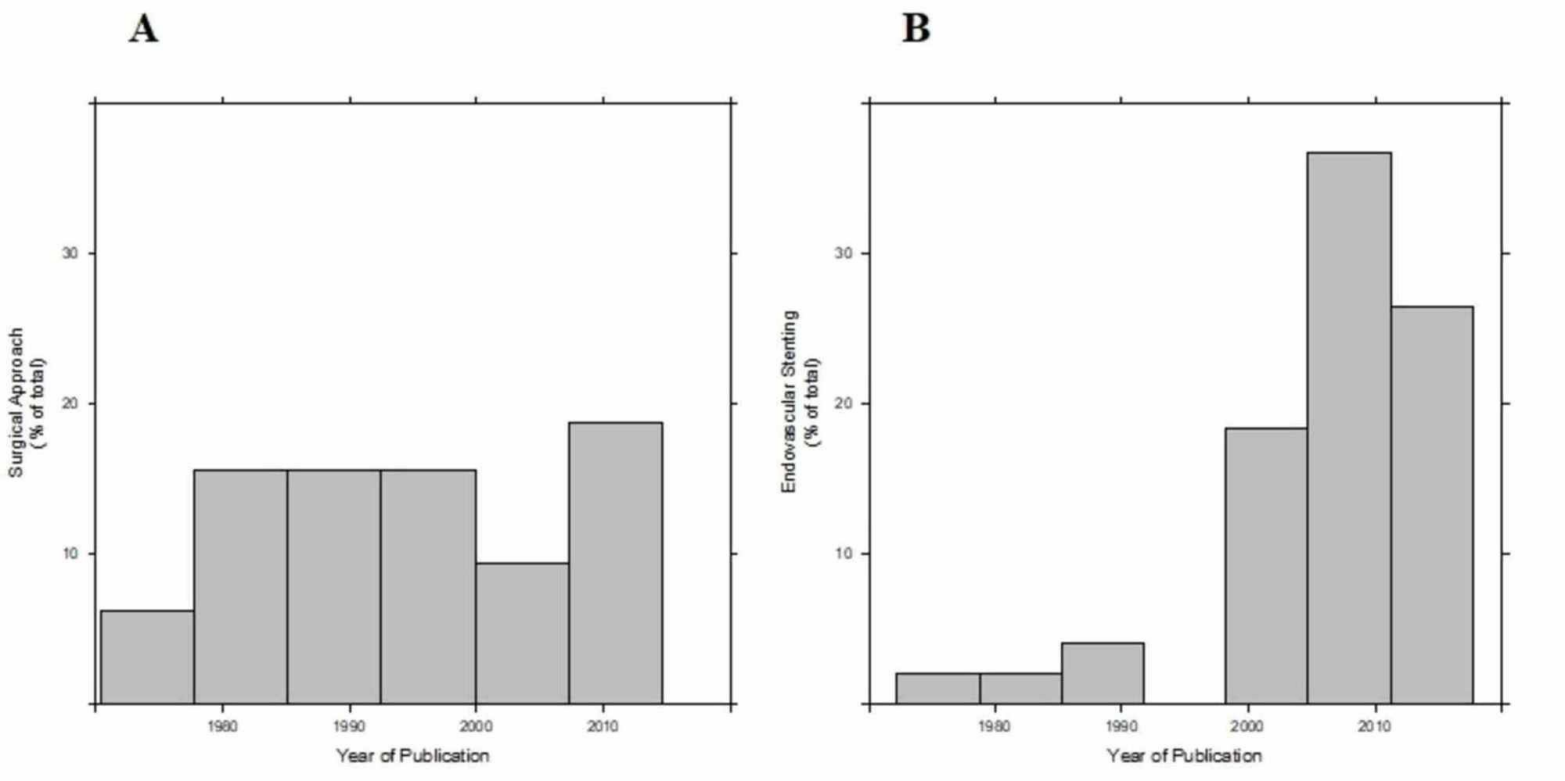
Histograms showing that the surgical approach was performed almost constantly throughout the years (3A; average year 1995; SD 13 years) whereas the endovascular treatment was more favorable during subsequent years (3B; average year 2006; SD 9 years). Per unequal variances t-test, the surgical approach was performed on average 11 years earlier in comparison to the endovascular stenting (p-value < 0.001; 95% CI 5 years, 17 years).

There was neither an association between patients’ age and the year of publication (p=0.419, Figure 2) nor between gender and age (p=0.860). As shown in Figure 3, a surgical approach has been performed almost exclusively in the past whereas an endovascular approach has become increasingly common within the last two decades. Endovascular management (35%) by stenting or embolization was utilized more frequently than the surgical approach (21%), which included bypass or nephrectomy. Most (56.6%) of the cohort was treated medically, with anticoagulation (45.2%) and/or with anti-hypertensive medical therapy (69%). With medical management, improvement of hypertension was noted in 90% of the cases but eight out of the 22 patients did ultimately require nephrectomy.

Discussion

To our knowledge, this is the first systematic review of cases of IRAD. The etiology of IRAD is not clearly defined. It has been associated with hypertension, atherosclerosis, connective tissue disorders (Marfan’s syndrome, fibromuscular dysplasia, Ehlers-Danlos syndrome, polyarteritis nodosa) [3-4]. IRAD has also been reported after inhaled cocaine use and during extreme physical exertion [5]. Spontaneous renal artery dissection has also been reported in healthy individuals [6]. Renal artery dissection, as shown in our review, is four times more prevalent in males than females. The reason for the disparity of the prevalence in males remains unknown. In this cohort, abdominal pain was the most common presenting symptom making the diagnosis challenging and separating this etiology from a gastrointestinal (GI) or genitourinary (GU) source difficult [7].

The presentation of IRAD could be acute or chronic. In the acute setting, IRAD is more likely to be spontaneous than due to trauma, iatrogenesis, or substance abuse. Chronic IRAD is usually silent or functional, and it is suspected that an underlying fibromuscular dysplasia could be the most frequent trigger for renal dissection [3-4,8]. Chronic dissections are typically found during investigation for renovascular hypertension (functional chronic IRAD) or gastrointestinal and genitourinary conditions that mimic IRAD. The true incidence or prevalence of chronic IRAD is difficult to accurately estimate because occasionally, renal artery dissection spontaneously resolves and may go undetected (silent chronic IRAD).

The pathogenesis of IRAD is not completely elucidated. Three mechanisms are proposed for renal artery dissection: shear stress, rupture of the vasa vasorum, and segmental arterial mediolysis (SAM). Connective tissue disorders, such as Ehlers Danlos and Marfan syndromes, have inherited defects to proteins involved in maintaining vascular integrity. In cases such as physical activity, trauma, cocaine use, and uncontrolled hypertension, shear stress can cause dissection in patients who have predisposing connective tissue disorder. Fibromuscular dysplasia and vasculitis like Kawasaki syndrome and polyarteritis nodosa have a predisposition to vasa-vasorum rupture. This then results in intramural hematoma, which causes medial ischemia and in sequence weakens the vessel wall leading to dissection [9]. Finally, SAM is an acute, non-inflammatory, non-atherosclerotic arteriopathy, which could lead to the dissection of renal arteries and other medium-sized visceral arteries [10]. The hallmark of SAM is arterial mediolysis, which refers to the vacuolar degeneration of arteria media. This leads to the creation of a gap between the media and adventitia layers where the dissecting hematoma and aneurysm occur. It is suspected that recurrent vasoconstrictive responses in the splanchnic vascular bed are the underlying trigger.

**Figure 4:**
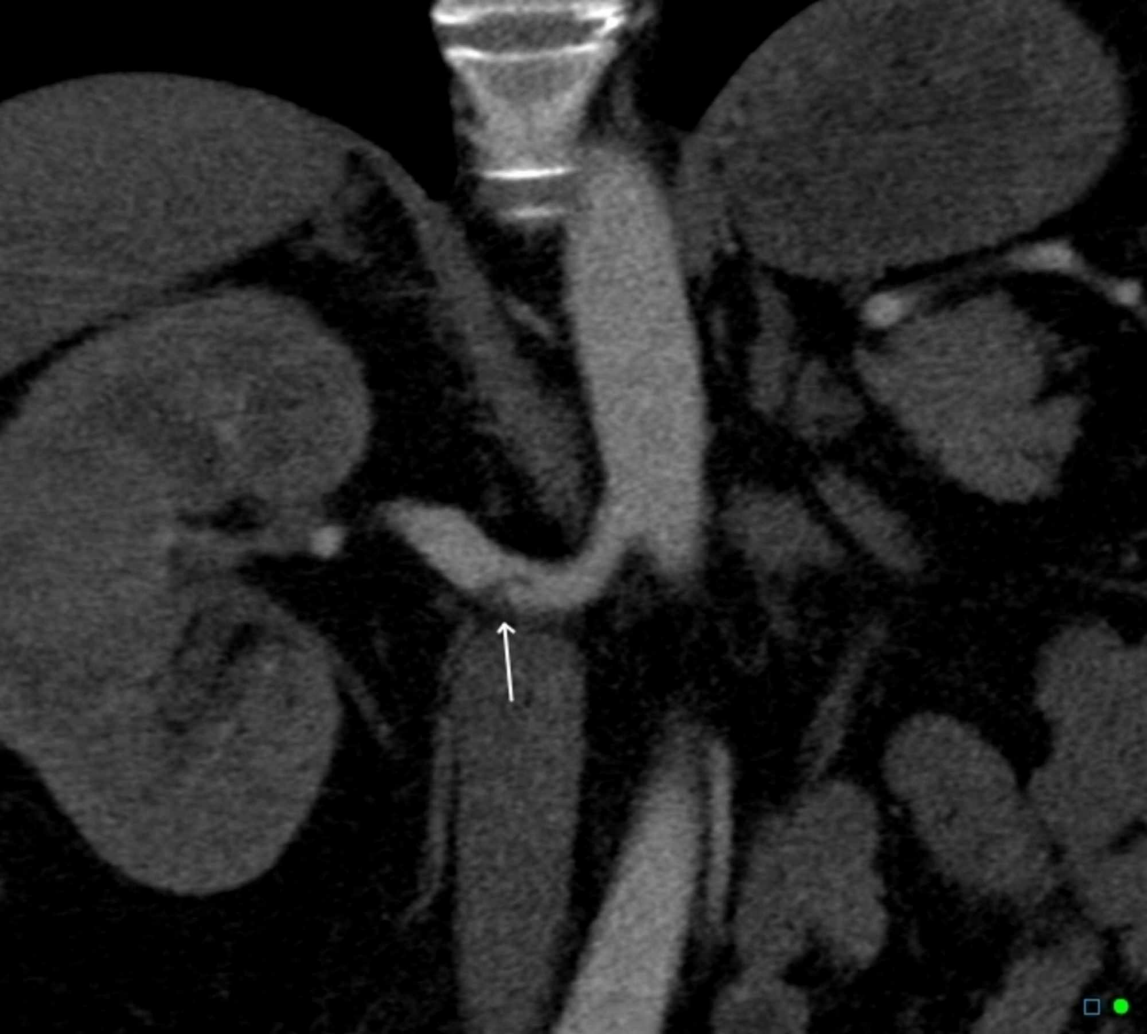
Coronal view of computed tomography angiography (CTA) showing right renal artery dissection - white arrow Figure courtesy: Dr Chris O'Donnell; https://radiopaedia.org/; from the case https://radiopaedia.org/cases/41977

**Figure 5:**
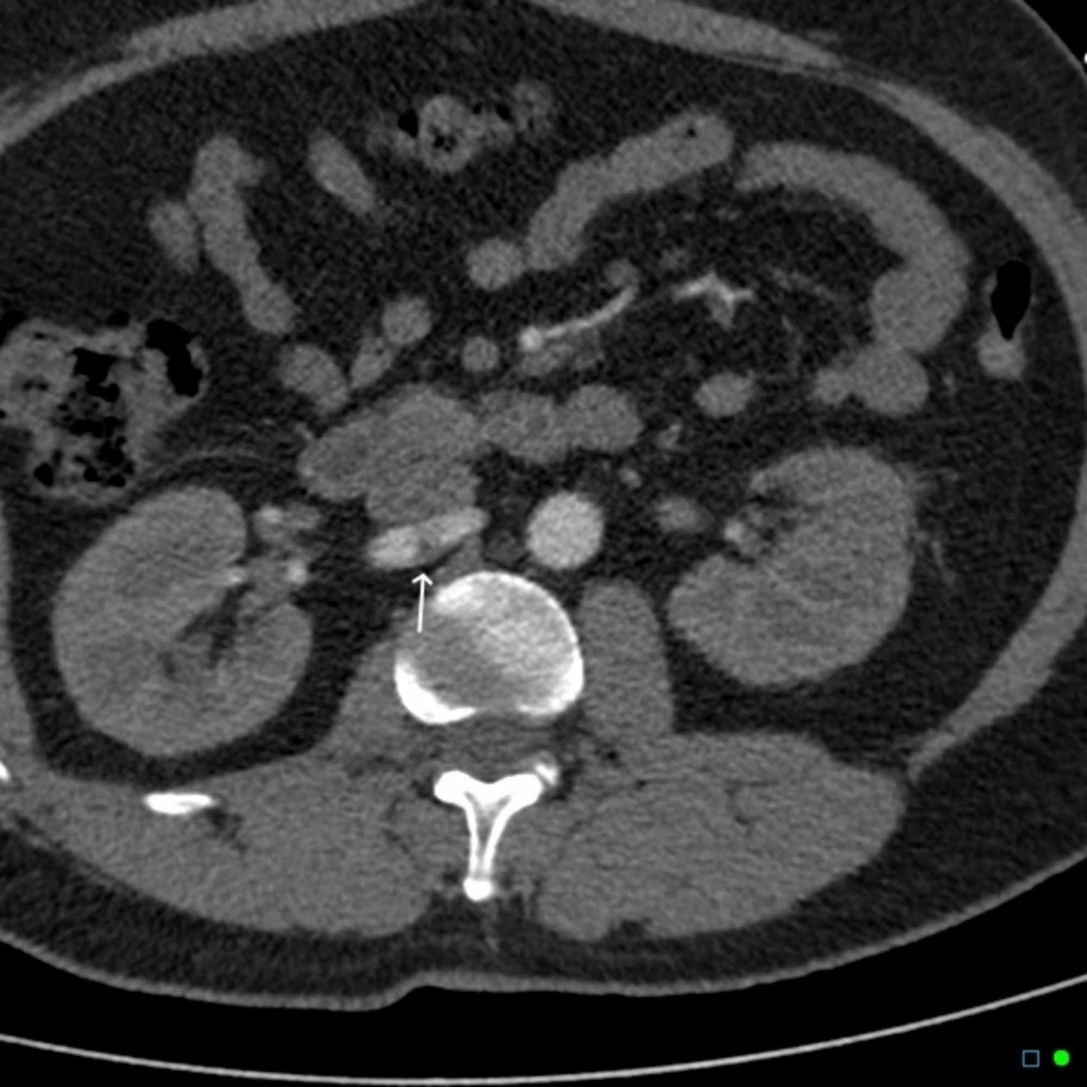
Transverse view computed tomography angiography (CTA) showing right renal artery dissection again - white arrow Figure courtesy: Dr Chris O'Donnell; https://radiopaedia.org/; from the case https://radiopaedia.org/cases/41977

The diagnosis of renal artery dissection is challenging. Four decades ago, almost all diagnoses of renal artery dissections were made at autopsy. However, with the advances made in imaging modalities, diagnoses are being made more frequently. The optimal imaging modality for the diagnosis of IRAD is unclear but from this review, it appears that CT and MRI have better diagnostic sensitivity than Doppler ultrasound and X-ray (Figure 4 and Figure 5) [11]. Renal angiography is a two-dimensional imaging modality that can assess for luminal narrowing, but it does not necessarily provide an accurate assessment of the arterial wall. Its limitations are evident in vessel sizing and the inability to clearly see the ostium in the anteroposterior view. Intravascular ultrasound or optical coherence tomography are better tools for the assessment of the arterial wall structure. These are frequently used in coronary artery wall assessment for spontaneous or iatrogenic dissection. Their utility is prominent in tissue characterization, differentiating between true and false lumen, accurate estimation for cross-sectional area, and the characterization of stent placement. Intravascular ultrasound (IVUS) may also help in delineating atherosclerosis and fibromuscular dysplasia (FMD) [2]. The potential for risks for instrumentation in this patient population makes catheter-based angiography not without inherent risks. Furthermore, the ability to perform an intervention is difficult, as traversing the dissection can be difficult without entering the false lumen. CTA and MRI avoid this inherent risk of catheter-based imaging.

This review demonstrates that both supportive medical treatment and interventional procedures (endovascular or surgical) have comparable outcomes. Conservative management refers to the use of antihypertensives, systemic anticoagulation, and pain control. Calcium channel blockers, angiotensin-converting enzyme inhibitors, and angiotensin receptor blockers are the more frequently used anti-hypertensives [6-12]. The rationale of using anticoagulation is to prevent the development of thrombosis at the site of endothelial injury, which can result in intramural thrombus formation that can propagate the dissection. Most of the experience published so far has been with vitamin K antagonists, which are typically bridged initially with heparin. The optimal duration of anticoagulation is unclear and there is a lack of data on the use of the oral anticoagulants in IRAD. Improvement of renal function post thrombolysis in IRAD has been described [13]. With advances in endovascular therapy, stenting, or coiling have also emerged as possible options for IRAD management [14]. Renal salvage is important when patients present with complicated renal failure, hemodynamic instability, and/or renovascular hypertension refractory to medical therapy. Operative repair, which for the most part involves nephrectomy, is challenging and should be performed only at institutions with experienced surgeons. So far, there is no guideline available for the treatment of IRAD [15-16].

## Conclusions

IRAD is an important and under-recognized condition, which is difficult to diagnose and even more challenging to treat. As the patient population is generally young and healthy with minimal comorbidities, the chances of missing this diagnosis is very high. A high degree of clinical suspicion is required to diagnose isolated renal artery dissection. CTA/MRI are the gold standard noninvasive diagnostic methods. The treatment of IRAD is challenging. There is a lack of proper guidelines regarding treatment. Generally, the outcome is favorable even in cases that received medical management. Surgical management is mostly used as salvage therapy. Further clinical trials can guide us through risk factors and treatment modalities.
